# The importance of identity-by-state information for the accuracy of genomic selection

**DOI:** 10.1186/1297-9686-44-28

**Published:** 2012-08-31

**Authors:** Tu Luan, John A Woolliams, Jørgen Ødegård, Marlies Dolezal, Sergio I Roman-Ponce, Alessandro Bagnato, Theo HE Meuwissen

**Affiliations:** 1 Department of Animal and Aquacultural Sciences, Norwegian University of Life Sciences, Ås, N-1432, Norway; 2 The Roslin Institute (Edinburgh), Royal (Dick) School of Veterinary Studies, University of Edinburgh, Roslin, Midlothian, EH25 9PS, UK; 3 Nofima, Ås, N-1432, Norway; 4 Università degli Studi di Milano, Dipartimento di Scienze e Tecnologie Veterinarie per la Sicurezza Alimentare, Via Celoria 10, Milano, 20133, Italy; 5 Instituto Nacional de Investigaciones Forestales Agrícolas y Pecuarias. C.E. Valles Centrales - CIRPAS, Melchor Ocampo 7, Etla, Oaxaca, 68200, México

## Abstract

**Background:**

It is commonly assumed that prediction of genome-wide breeding values in genomic selection is achieved by capitalizing on linkage disequilibrium between markers and QTL but also on genetic relationships. Here, we investigated the reliability of predicting genome-wide breeding values based on population-wide linkage disequilibrium information, based on identity-by-descent relationships within the known pedigree, and to what extent linkage disequilibrium information improves predictions based on identity-by-descent genomic relationship information.

**Methods:**

The study was performed on milk, fat, and protein yield, using genotype data on 35 706 SNP and deregressed proofs of 1086 Italian Brown Swiss bulls. Genome-wide breeding values were predicted using a genomic identity-by-state relationship matrix and a genomic identity-by-descent relationship matrix (averaged over all marker loci). The identity-by-descent matrix was calculated by linkage analysis using one to five generations of pedigree data.

**Results:**

We showed that genome-wide breeding values prediction based only on identity-by-descent genomic relationships within the known pedigree was as or more reliable than that based on identity-by-state, which implicitly also accounts for genomic relationships that occurred before the known pedigree. Furthermore, combining the two matrices did not improve the prediction compared to using identity-by-descent alone. Including different numbers of generations in the pedigree showed that most of the information in genome-wide breeding values prediction comes from animals with known common ancestors less than four generations back in the pedigree.

**Conclusions:**

Our results show that, in pedigreed breeding populations, the accuracy of genome-wide breeding values obtained by identity-by-descent relationships was not improved by identity-by-state information. Although, in principle, genomic selection based on identity-by-state does not require pedigree data, it does use the available pedigree structure. Our findings may explain why the prediction equations derived for one breed may not predict accurate genome-wide breeding values when applied to other breeds, since family structures differ among breeds.

## Background

Substantial advances in genotyping technology have been achieved over the past decade. With the availability of genome-wide, dense molecular markers, genomic selection (GS) has now become practical and its effectiveness in dairy cattle breeding has been demonstrated in many countries [1-6]. In this approach, genome-wide breeding values (GW-EBV) are predicted through the use of dense markers covering the whole genome [7]. It differs from traditional breeding value estimation, which uses only phenotypic data and pedigree information. Availability of marker genotypes for many thousands of loci across the whole genome allows GS to predict genetic value more precisely than traditional selection methods [8].

The basic principle of GS is that, given a sufficiently high marker density, each quantitative trait locus (QTL) is in linkage disequilibrium (LD) with a number of nearby markers, and a high fraction of the genetic variance is expected to be explained by these markers. Habier *et al.*[9] found that accuracies of GW-EBV also incorporate information on LD arising from recent family structures. The fact that this LD generated by family structure can be explained by linkage analysis (LA) implies that GS can also use LA information. A genomic identity-by-descent (IBD) matrix, containing identity-by-descent probabilities within the known pedigree, depicts this LA information. In addition, information from identical-by-state (IBS) markers may provide LD information among the founders of the pedigree, since the markers may be shared through older common ancestors than those included in the known pedigree. This LD information is equivalent to allowing non-zero genetic covariance among founders for the traits of interest. Since mutations occurred on average 2*N*_*e*_ generations ago, where *N*_*e*_ is the effective population size, IBS can consider relationships up to 2*N*_*e*_ generations back in time, while IBD takes into account only generations back to the founders of the known pedigree. The implications of these studies are that GS combines information on LD among founders and relationships between known relatives (IBD relationships).

When genomic selection applies an IBS derived relationship matrix, it relies on the assumption that the relationships between individuals at the marker level reflect to a large extent their relationships also at the QTL level [8]. For low-density marker panels, the accuracy of the resulting GW-EBV would therefore be expected to be reduced compared to using high-density panels, since the relationships at the marker level would be an imperfect estimator of relationships at the QTL level [10]. However, even for highly dense marker maps, LD between marker and QTL alleles may be imperfect, due to the fact that QTL and marker (SNP) mutations in the population may be of different age [11]. If the flanking SNP markers are older than a closely linked QTL mutation, similarity at the marker level will be an imperfect indicator of similarity at the QTL level. Similarly, if the QTL mutation is older than the flanking marker alleles, individuals with different marker alleles may still share closely linked QTL alleles. However, among close relatives, the close linkage between marker and QTL alleles implies that genomic similarity at the marker level will closely reflect similarity at the QTL level. Hence, one option for genomic evaluation is to combine genomic and pedigree information to estimate IBD relationships across all loci, using linkage analysis. Here, animals will be regarded as related to the extent that identical marker alleles can be traced back to a common ancestor.

The objective of this study was to investigate the accuracy of GW-EBV prediction using IBS relationships based on (population-wide) LD and using genomic IBD relationships based on a limited number of generations within a known pedigree, and how much IBS information can improve the accuracy of GW-EBV over and above the use of IBD information. Genomic evaluations were conducted on deregressed estimated breeding values for milk traits of progeny tested Italian Brown Swiss bulls using IBS and IBD information. Linkage analysis was performed at the marker positions in order to estimate the IBD relationships. Accuracy of the GW-EBV was assessed by replicated cross-validation.

## Methods

### Genotypic and phenotypic data

**Figure 1:**
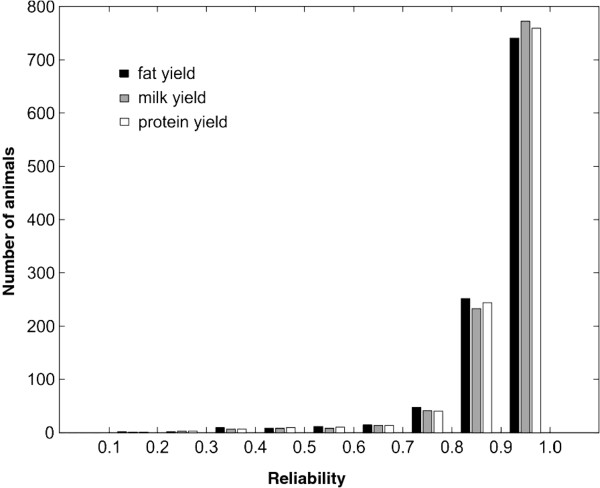
Histogram of the reliabilities of the deregressed proofs.

One thousand and eighty six Italian progeny-tested Brown Swiss bulls were genotyped with the *Illumina* BovineSNP50 BeadChip, which included 51 582 single nucleotide polymorphism (SNP) markers. A total of 35 706 SNPs remained after removing SNP with minor allele frequency (MAF) < 0.05 and those that failed the test of missing genotypes (>5 %). The phenotypic data of the 1086 progeny-tested bulls were conventional estimated breeding values (EBV) for the following traits: kilograms of milk yield, kilograms of milk fat yield and kilograms of milk protein yield. The EBV were deregressed [12] to be used as response variables and will be referred to as Deregressed Proofs (DP). A histogram of the reliabilities of the DP is given in Figure 1. About 92% of the reliabilities were very high, i.e. >0.8, which implies that the DP were close to the true breeding values of the bulls.

### Cross-validation

To obtain test datasets for cross-validation, the phenotypes of a defined number of individuals were masked, i.e., by defining their phenotype as “unknown”. Six non-overlapping cross-validation datasets were created by randomly selecting 181 bulls at a time, without replacement, i.e., every phenotype was masked precisely once. The DP of the masked individuals were predicted by the GS methods (see next section). For each of the six cross-validation sets, the correlation coefficient between the 181 predicted GW-EBV and DP was calculated and squared to be used as a measure of the reliability of the EBV predictions from GS. In order to obtain standard errors, the division into sets and all GW-EBV predictions were replicated six times.

### GW-EBV prediction based on genomic IBS relationships

The model used in the study to predict GW-EBV with IBS information (i.e. based solely on LD) is best linear unbiased prediction (G-BLUP). A model equivalent to that described in the literature [9,13] was used, where individual animal effects are fitted together with a genomic relationship matrix, instead of individual marker effects. The model can be expressed as:

y=1μ+Za+e

where **y** is a vector of phenotypes for the traits; *μ* is the overall mean; ***Z*** is a *M*_*y*_ × *M* design matrix linking the animals to the records, where *M* (*M*_*y*_) is the number of bulls (with records); ***a*** is a *M* × 1 vector of genetic effects of the animals and ***e*** is the random residual vector. It is assumed that ***a*** ~ *N* (**0**, **G**_**IBS**_*σ*_*a*_^*2*^) where *σ*_*a*_^*2*^ is additive genetic variance, **G**_**IBS**_ is the genomic relationship matrix based on IBS markers. GW-EBV of animals without records were calculated by including them in the ***a*** vector (and **G**_**IBS**_), but not linking the animal effect to a record in matrix ***Z***, such that the solution to the mixed model equations also yields EBV for animals without records.

To construct IBS relationships, let *X*_*ij*_ denote the “standardized” genotype of animal *i* for SNP *j*, i.e., $X_{\mathrm{ij}}=\left(g_{\mathrm{ij}}-2p_{j}\right)/\sqrt{2p_{j}\left(1-p_{j}\right)}$ where *g*_*ij*_ is the genotype of animal *i* and SNP *j*, with *g*_*ij*_ = 0, 1 or 2 where SNP genotypes are “0 0”, “1 0” or “1 1”, respectively, and *p*_*j*_ is the allele frequency of SNP *j*. Standardization is such that the mean is zero and the variance of *X*_*ij*_ is 1 [8]. Then **G**_**IBS**_ is the covariance matrix of the standardized marker genotypes, which is calculated as **G**_**IBS**_ = **XX’/N**_**m**_, where **N**_**m**_ is the number of markers. **G**_**IBS**_ was inverted, and BLUP was used to predict EBV of masked and non-masked individuals. The model was implemented by using the package ASReml [14].

### GW-EBV prediction based on genomic IBD relationships

Using a standard animal model

y=1μ+Zu+e

the same trait phenotypes **y** can be described by the overall mean *μ*, the *M*_*y*_ × *M* incidence matrix ***Z***, a *M* × 1 vector of additive genetic effects of individuals ***u***, and a random residual vector ***e***. It is assumed that ***u*** ~ *N* (**0**, **G**_**IBD**_*σ*_*u*_^*2*^), where **G**_**IBD**_ is the LA based genomic IBD relationship matrix. The **G**_**IBD**_ matrix assumes that the founders of the pedigree are non-IBD and that all the IBD is due to common ancestors within the known pedigree.

In order to estimate the **G**_**IBD**_ matrix, first, the LDMIP method (Linkage Disequilibrium Multilocus Iterative Peeling) [15], was used to estimate the probability that an offspring inherits the paternal/maternal allele from its sire, and similarly the probability it inherits the paternal/maternal allele from its dam. Five or more generations of pedigree were available for the iterative peeling for all bulls. The probability of maternal inheritance was equal to 1 minus the probability of paternal inheritance. Secondly, the probabilities of paternal inheritance were used to set up an IBD matrix, **G**_**IBD,***j*_, at every marker position *j*, using Fernando and Grossman’s rules [16]. Third, the **G**_**IBD,***j*_ matrices were averaged across all marker loci, to obtain an overall IBD relationship matrix, **G**_**IBD**_. The inverse of **G**_**IBD**_ was then used by ASReml to predict GW-EBV of both phenotyped and non-phenotyped individuals.

### GW-EBV prediction based on genomic IBS + IBD relationships

Prediction of EBV using both IBS and IBD information can be expressed as:

(3) y=1μ+Za+Zu+e

where it is assumed that ***a*** ~ *N* (**0**, **G**_**IBS**_*σ*_*a*_^*2*^) and ***u*** ~ *N* (**0**, **G**_**IBD**_*σ*_*u*_^*2*^), with the IBS and IBD based genomic relationship matrices **G**_**IBS**_ and **G**_**IBD**_ defined as described above. ASReml was used to predict breeding values and estimate *σ*_*a*_^*2*^, *σ*_*u*_^*2*^ and *σ*_*e*_^*2*^. Subsequently, GW-EBV were calculated as$EBV=\hat{a}+\hat{u}$, where $\hat{a}$and $\hat{u}$ are vectors of predicted breeding values associated with **G**_**IBS**_ and **G**_**IBD**_, respectively.

## Results

### Reliability of GW-EBV prediction

Table 1 shows the reliability, i.e. the square of the accuracy of the GW-EBV prediction, by using only IBS information, IBD information, and IBS + IBD information. The table shows the mean and the standard error of the predictive reliability obtained for the 181 masked individuals when the 905 non-masked animals were in the training set. The mean is an average of 36 values, six replicates of random division of the bulls into six sets. The reliabilities were similar for all three methods and for all traits studied. The reliability of the GW-EBV prediction using IBS + IBD information was virtually the same as that using IBD information alone, indicating that combing IBS and IBD information yields hardly any improvement.

**Table 1:** Reliability of GW-EBV (±SE, based on 36 calculations) obtained using IBS, IBD, and IBS + IBD genomic relationship matrices

**Methods**	**Fat yield**	**Milk yield**	**Protein yield**
IBS	0.5901 (± 0.0165)	0.5832 (±0.0164)	0.6126 (±0.0159)
IBD	0.6000 (± 0.0218)	0.6013 (±0.0203)	0.6285 (±0.0207)
IBS + IBD	0.6035 (± 0.0205)	0.6034 (±0.0195)	0.6308 (±0.0199)

### Effect of the number of generations used

To investigate the effect of the number of generations in the pedigree, which defines the base generation with non-IBD individuals, on the reliability of the GW-EBV with IBD information, we performed iterative peeling using 1, 2, 3, 4 and 5 generations of pedigree data, respectively. The corresponding **G**_**IBD**_ matrix was inverted and used by ASReml for the EBV prediction. Table 2 shows that reliability decreased when less than 3 generations of pedigree data were used, and the reliability of the prediction increased only very slightly when the number of generations of pedigree used was greater than 3.

**Table 2:** Reliability of EBV (±SE, based on 36 calculations) predicted with IBD information using different numbers of generations of pedigree data

**Number of generations**	**Fat yield**	**Milk yield**	**Protein yield**
1	0.5304 (± 0.0257)	0.5488 (± 0.0245)	0.5714 (± 0.0253)
2	0.5765 (± 0.0248)	0.5866 (± 0.0236)	0.6105 (± 0.0241)
3	0.5875 (± 0.0240)	0.5915 (± 0.0226)	0.6154 (± 0.0231)
4	0.5923 (± 0.0234)	0.5949 (± 0.0219)	0.6194 (± 0.0223)
5	0.5923 (± 0.0230)	0.5936 (± 0.0214)	0.6184 (± 0.0218)

### Variance components, regression and other results

Table 3 shows variance components estimated by ASReml using IBS, IBD and IBS + IBD information. Results suggest that the IBD matrix explained substantially more variance than the IBS matrix, when they were fitted together in the model. Table 4 presents coefficients of regression (mean and standard error) of DP on the GW-EBV predicted using only IBS information, IBD information and IBS + IBD information, and on the GW-EBV predicted with IBD using 1, 2, 3, 4 and 5 generations of pedigree data, based on six replicates of six training datasets. The regression of the DP on the GW-EBV predicted using IBD information was higher than that on the GW-EBV predicted using IBS information. The regression coefficients were generally slightly higher than 1, which suggests that the variance of the GW-EBV was slightly too low relative to the variance of the DP.

**Table 3:** Mean of variance components estimated using IBS, IBD, and IBS + IBD genomic relationship matrices

**Methods**	**Fat yield**	**Milk yield**	**Protein yield**
IBS			
*σ*_*a*_^*2*^	1010.37	527771	649.61
*σ*_*e*_^*2*^	149.49	93358	104.86
IBD			
*σ*_*u*_^*2*^	1020.94	558117	671.47
*σ*_*e*_^*2*^	2.99	8716	12.56
IBS + IBD			
*σ*_*a*_^*2*^	105.26	48690	56.09
*σ*_*u*_^*2*^	1000.62	548435	660.88
*σ*_*e*_^*2*^	3.04	8604	12.53

*σ*_*a*_^*2*^ and *σ*_*u*_^*2*^: estimated genetic variances using IBS and IBD relationship matrix, respectively; *σ*_*e*_^*2*^: mean error variance.

**Table 4:** Coefficients of regression (±SE, based on 36 calculations) of deregressed proofs on GW-EBV obtained using IBS + IBD, IBS, and IBD genomic relationship matrices and using IBD relationships obtained from different numbers of generations of pedigree data

**Methods**	**Fat yield**	**Milk yield**	**Protein yield**
IBS + IBD	1.1472 (±0.0279)	1.1411 (±0.0235)	1.1409 (±0.0224)
IBS	1.0462 (±0.0365)	1.0533 (±0.0291)	1.0519 (±0.0286)
IBD	1.1434 (± 0.0268)	1.1391 (±0.0230)	1.1390 (±0.0219)
IBD using number of generations			
1	1.1354 (± 0.0150)	1.1428 (±0.0135)	1.1376 (±0.0122)
2	1.1257 (±0.0190)	1.1215 (±0.0159)	1.1174 (±0.0146)
3	1.1271 (±0.0208)	1.1198 (±0.0174)	1.1166 (±0.0164)
4	1.1318 (±0.0227)	1.1248 (±0.0188)	1.1227 (±0.0180)
5	1.1331 (±0.0236)	1.1255 (±0.0197)	1.1239 (±0.0189)

The means of the correlations between the GW-EBV using IBD information and GW-EBV using IBS information, for six replicates of six training datasets, were 0.959, 0.955 and 0.959 for fat, milk and protein traits, respectively. Thus, the GW-EBV obtained using IBD versus IBS information were somewhat different, although their reliabilities were very similar, suggesting that the information comes from slightly different sources. The correlation between the elements of the **G**_**IBS**_ and **G**_**IBD**_ matrices was 0.959, which agrees well with the differences in GW-EBV.

## Discussion

In our data there was no evidence that IBS information improves the accuracy of selection or the fit of the model, when the model already contains four or more generations of IBD information. A number of factors can have caused or contributed to this finding, which will be discussed below. We believe that the most important factor is that recent family relationships are strong in our bulls population and that older, more distant relationships contribute little to the accuracy of selection. If this is the case, our result would also apply to other populations with strong recent family relationships. In the next paragraph, we explain why we believe that other factors are less important.

The estimates of the variance components in Table 3 show that the IBD matrix explained much more variance than the IBS matrix when they were fitted together in the model. This could be due to (1) the IBD matrix being more accurately estimated than the IBS matrix, although 35 K markers were used for both matrices; and (2) the relationships further back in time, which are not depicted by IBD, are not important for explaining covariances between records. In addition, estimates of the residual variance (*σ*_*e*_^*2*^) were substantially lower when IBD information was used in the model. This may be explained by (1) the IBD and IBD + IBS model overfitted the data; or (2) the IBS model did not explain all the genetic variance which increased the estimate of *σ*_*e*_^*2*^. In order to distinguish between these two explanations, we also estimated the variance components using a pedigree-based relationship matrix, which is known to yield unbiased estimates. The resulting *σ*_*e*_^*2*^ estimates were 4.21, 11 603, and 16.4 for fat, milk and protein yield, respectively. These estimates are close to those of the IBD and IBD + IBS model, suggesting that explanation (2) is more likely than (1), although the IBD and IBD + IBS model seem also to overfit the data a little (since their estimates of *σ*_*e*_^*2*^ were somewhat lower than those of the pedigree based model).

A possible explanation for the too high regression coefficients in Table 4 is that the models overfit the data. However, when fitting a pedigree-based relationship matrix, the regression coefficients were about 1.17 for all three traits (results not shown elsewhere) and thus similar to those of the IBD and IBD + IBS models. Since the pedigree-based model is expected to be unbiased, these biases seem to be due to the deregressed EBV data rather than due to the models being used. Possibly the coefficient used to perform the deregression when calculating the deregressed EBV was too high, resulting in these inflated regression coefficients.

It may be postulated that the Italian Brown Swiss population is perhaps rather homogeneous, and that our results cannot be generalized to other populations with more population stratification, e.g. due to a recent admixture of populations. However, this would require that the population admixture took place just before the pedigree recording started, because if the population admixture occurred more than eight or more generations ago, the contributions of the founder populations would have converged and be the same for every bulls. In this case, genetic differences between bulls could not be explained by differential contributions of the founder populations.

In the present study, we randomly selected 181 bulls at a time without replacement, to produce six non-overlapping cross-validation datasets. This cross-validation yields a statistically valid estimate of accuracy and predicts the accuracy of a random bulls that could have been in the training dataset but was not. In practical animal breeding, the prediction of young bulls is however more relevant. This requires the prediction of bulls which do not represent a random sample of the training data, and whose genotypes systematically deviate from those of the training bulls, which thus requires an extrapolation from the training data. We did not attempt such an extrapolation here, because the number of young bulls was rather small and included only one set of young bulls. This would not have allowed us to replicate results, which would have made it impossible to calculate standard errors and compare accuracies for statistically significant differences.

The IBS + IBD model was not more accurate than the IBD model, which may be due to the 35 k SNP chip not showing a perfect LD with all the genes, and thus that a part of the genetic variance is not accounted for [15]. The genetic variance that was not picked up by IBS information is explained by the **G**_**IBD**_ matrix, since it focuses on within-family linkage analysis, which may have caused the IBD information to yield slightly higher accuracy than the IBS information.

The IBD genomic relationship matrix, **G**_**IBD**_, shows the relationships since a defined base generation, in which animals are assumed unrelated. The **G**_**IBD**_ matrix estimates the probability of IBD only based on the pedigree and the inheritance of marker alleles through the pedigree (linkage analysis). Marker alleles that are IBS are not necessarily IBD, unless they can be traced back to a common ancestor within the pedigree. The IBS genomic relationship, **G**_**IBS**_, shows the genomic similarity between two animals based on the markers being IBS, which also depicts relationships before the base generation of the pedigree. Therefore, the IBS genomic relationship matrix can be regarded as including many more, up to 2*N*_*e*_, generations of pedigree. GS relies on marker information to predict breeding values and hence in general, the **G**_**IBS**_ matrix is used for GS based on the BLUP method. In this study, we used real data to show that using IBD information from a few recent generations can achieve similar accuracy of GS as using IBS information from the markers. Since the accuracies only marginally improve when including IBD information in a model that already contains IBS information, the IBS information alone, and thus LD information, is capable of recovering a large part of the IBD information.

The amount of IBD information that can be recovered by the markers may depend on marker density. Table 2 suggests that most of the information in genomic selection using 35 K SNP in the study came from the last four generations of data. It could be that a higher marker density reduces errors in the estimates of relationship between distant relatives, which may improve the contribution of ancient relationships to the accuracy of GW-EBV. However, prediction of GW-EBV using IBD relationships depends less on marker density, since closely related animals usually share larger chromosomal segments, which may be accurately identified even with a sparse marker map. Thus, the IBD matrix approach may achieve the same accuracy with a less dense SNP panel.

Magnitude of LD, and thus reliability of GS, depend on the effective population size [17]. However, the effective population size varies with time. Our results show that the very recent population structure is critical for the accuracy of GS since the use of four or more generations of pedigree data to calculate IBD genomic relationships gave similar (or even slightly better) accuracy than using **G**_**IBS**_. The results also suggest that the recent effective population size is most relevant for the prediction of the reliability of GS with the current SNP densities ~30 – 50 K. The question of how important IBS is depends on the family structure of the population: IBS is less important if recent family relationships are strong, as is usually the case in dairy bulls populations, and was the case here. With increasing SNP density, it is possible to better capture LD with QTL since the expected distance between QTL and flanking marker loci becomes smaller (The Bovine Hapmap Consortium [18]), and the estimation error of distant genomic relationships decreases. This is expected to increase the importance of the IBS contribution to accuracy and consequently increase the importance of the historic effective population sizes.

## Conclusions

The results show that the accuracy of GW-EBV obtained by IBD relationships, estimated through linkage analysis using four or more generations of pedigree and marker information of the bulls, cannot be improved by including IBS information. This is most likely because the recent family structure in our dairy bulls population was so dominant that more distant relationships became less important. Possibly, the distant genomic relationships were also too inaccurately estimated by the 35-50 K SNP. Although GS based on IBS does in principle not require pedigree data, it does use the available population structure, which is indirectly included through the IBS marker information. Our findings may explain why the prediction equations derived in one breed may not predict accurate GW-EBV when applied to other breeds [18], because the information derived from the family structure is not relevant for the other breeds.

## Competing interests

The authors declare that they have no competing interests.

## Authors' contributions

TL performed the study and drafted the manuscript. JAW contributed to the draft writing. JØ contributed to the draft writing and revised the manuscript critically. MD, SIRP, AB prepared the genotypic and phenotypic data. THEM planned and coordinated the whole study, and contributed to the manuscript writing. All the authors read and approved the final manuscript.
